# Solubility enhancement of mefenamic acid by inclusion complex with β-cyclodextrin: *in silico* modelling, formulation, characterisation, and *in vitro* studies

**DOI:** 10.1080/14756366.2020.1869225

**Published:** 2021-02-08

**Authors:** Dounia Sid, Milad Baitiche, Zineb Elbahri, Ferhat Djerboua, Mokhtar Boutahala, Zouhair Bouaziz, Marc Le Borgne

**Affiliations:** a Département de Génie des Procédés, Faculté de Technologie, Laboratoire de Préparation, Modification et Applications des Matériaux Polymériques Multiphasiques, Université Ferhat Abbas Sétif-1, Sétif, Algérie; b Faculty of Exact Sciences, Laboratory of Materials and Catalysis, Djillali Liabès University of Sidi Bel Abbès, Sidi Bel Abbès, Algeria; c Département de Génie des Procédés, Faculté de Technologie, Laboratoire de Génie des Procédés Chimiques, Université Ferhat Abbas Sétif-1, Sétif, Algérie; d EA 4446 Bioactive Molecules and Medicinal Chemistry, SFR Santé Lyon-Est CNRS UMS3453 – INSERM US7, Université Claude Bernard Lyon 1, Univ Lyon, Lyon, France; e Centre de Recherche en Cancérologie de Lyon, Centre Léon Bérard, Small Molecules for Biological Targets Team, CNRS 5286, INSERM 1052, Université Claude Bernard Lyon 1, Univ Lyon, Lyon, France

**Keywords:** Mefenamic acid, β-cyclodextrin inclusion complexes, solubility, characterisation, in vitro studies

## Abstract

The aim of this study was to prepare and characterise inclusion complexes of a low water-soluble drug, mefenamic acid (MA), with β-cyclodextrin (β-CD). First, the phase solubility diagram of MA in β-CD was drawn from 0 to 21 × 10^−3^ M of β-CD concentration. A job’s plot experiment was used to determine the stoichiometry of the MA:β-CD complex (2:1). The stability of this complex was confirmed by molecular modelling simulation. Three methods, namely solvent co-evaporation (CE), kneading (KN), and physical mixture (PM), were used to prepare the (2:1) MA:β-CD complexes. All complexes were fully characterised. The drug dissolution tests were established in simulated liquid gastric and the MA water solubility at pH 1.2 from complexes was significantly improved. The mechanism of MA released from the β-CD complexes was illustrated through a mathematical treatment. Finally, two *in vitro* experiments confirmed the interest to use a (2:1) MA:β-CD complex.

## Introduction

The access of drugs to biological targets is essential for producing biological effect(s) in living organisms. Globally physicochemical properties and absorption, distribution, metabolism, elimination (ADME) parameters of a drug substance are essential to ensure the accessibility to target(s)^1^. Moreover, only the free part of the drug substance diffuses in tissues from the blood and then can access the active binding site of the desired target. Among all parameters to control for a successful treatment, an acceptable level of drug substance solubility is required. In the case of low solubility drug substances, inclusion complexes can be an interesting alternative to facilitate drug administration. Additionally, *in silico* methods were also developed to optimise this approach and then improve the use of low solubility drug substances^2^.

Actually, many efficiency issues caused by low solubility drugs are highlighted in recent works, for instance, according to Kalepu and Nekkanti^3^, more than 80% of drugs are sold as tablets, in which 40% have low water solubility. The same study illustrates more serious situation concerning R&D drug candidates, in which 90% could fail due to low solubility problems. However, to improve the dissolution profile of poorly soluble drugs, various approaches are proposed such as particle size reduction^4^, drug dispersion in carrier^5^, modification of crystal habit^6^, use of surfactant^7^, self-emulsifying formulations^8^, and complexation with cyclodextrins (CDs)^9^. This later is one of the frontier techniques that are employed in almost 56 pharmaceutical products^10^, where these oligomers obtained from enzymatic degradation of starch are exploited. These cycloamyloses are composed of D-glucopyranoside units linked by α(1 → 4)-glycosidic bonds. Typical native CDs involve six, seven, or eight glucose units, denoted α-, β- and γ-CDs, respectively. CDs belong to cage molecules family due to their hydrophobic cavity structure, and hydrophilic outer surface. Indeed, the most significant characteristic of CDs is their ability to form inclusion complexes with various molecules through host–guest interactions^11^^,^^12^. These inclusion complexes have been revealed to improve the apparent stability, solubility, dissolution rate, and bioavailability of the guest bioactive molecules^13–16^. Among various CDs, β-CD is the most frequently used in pharmaceutical excipient due to its wide availability, low cost, excellent biocompatibility, preferred cavity dimension, and wide regulatory acceptance^17^. We note that two other naturally occurring CDs (α- and γ-CDs) are more cost-effective compared to β-CD. Moreover, a recent report provided that a β-CD derivative known as the hydroxypropyl-β-CD, even though it has a better solubility than β-CD, is undesirable due to its toxicity^18^.

Mefenamic acid (MA), 2-[(2,3-dimethylphenyl)amino]benzoic acid, was selected as a drug model for our study. It is a potent non-steroidal anti-inflammatory drug (NSAID) of the anthranilic acid class. Moreover, it shows preferential inhibition of cyclo-oxygenase-2 (COX-2), inhibiting the action of prostaglandin synthetase^19–21^. Furthermore, MA is approved by the food and drug administration (FDA) in 1965^22^. Due to its low solubility in water and high permeability in the gastrointestinal (GI) tract, MA is classified in the biopharmaceutics classification system (BCS) as class II drug^23^^,^^24^. It is widely indicated for inflammatory diseases and also as an analgesic for the treatment of musculoskeletal, osteoarthritis, rheumatoid arthritis, menstrual symptoms, and headach^19^^,^^25–27^. Furthermore, MA has been shown to have therapeutic effects in neurodegenerative disease (e.g. Alzheimer disease)^28^. In addition, MA, like other anti-inflammatory drugs, is emerging as new chemopreventive agents against cancer^29^. However, due to its low water solubility, high doses (250 or 500 mg twice a day administration) and side effects, mainly related to GI adverse consequences, including bleeding, ulceration or colitis lesions, and steatorrhoea, which can be sometimes fatal, made the use of MA limited^19^^,^^30–32^.

On the other hand, the study of the formation and the stability of guest–host complexes can be assisted by molecular modelling methods, like docking and quantum mechanics (QM), which may mimic the behaviour of inclusion systems at the atomistic level^33^. These modelling methods have been playing an important role in providing 3 D simulation structures in order to understand the mechanism of CDs inclusion formation systems, assist the formulation design and simplify the formulation screening procedures followed by the delivery studies^34^^,^^35^.

In this study, we investigated the complexation of MA with β-CD for improving both solubility and dissolution rate. We were motivated by the fact that β-CD complexation enhanced the solubility of other acidic NSAIDs, including tolfenamic acid^36^, niflumic acid^37^, indomethacin^38^, and ketoprofen^39^. Some (1:1) complexes’ types of MA:β-CD have been previously developed by authors using precipitation^40^^,^^41^. or kneading (KN)^42^ methods In these studies, the molar concentration of β-CD did not exceed 10 mM, the solubility phase diagram was A_L_ type and so the proposed MA:β-CD complex ratio was 1:1 of MA and β-CD. In these previous works^40–42^, the Job’s plot method was not also investigated. In this work, phase solubility diagram of MA using increasingly high β-CD concentrations (from 0 to 21 × 10^−3^ M), job’s plot and molecular modelling simulation were associated to study MA:β-CD inclusion complexes. Three methods were carried out using solvent co-evaporation (CE), KN, and physical mixture (PM) for the preparation of these complexes. Then selective physicochemical determinations based on Fourier-transform infra-red spectroscopy (FTIR), differential scanning calorimetry (DSC), X-ray powder diffraction (XRPD), and scanning electron microscopy (SEM) were performed to characterise all complexes. *In vitro* aqueous solubility and dissolution rate profiles of the complexes were also performed. Finally, the resulting inclusion complexes were evaluated *in vitro* using protein denaturation and membrane stabilisation methods.

## Materials and methods

### Chemicals

MA, C_15_H_15_NO_2_, molecular weight: 241.29 g/mol was a gift from SALEM Laboratories El- Eulma, Algeria. β-CD (C_42_H_70_O_35_, molecular weight: 1134.00 g/mol) was purchased from Sigma-Aldrich, St. Louis, MO. All other chemicals used were of analytical grades. All reagents were used as received.

### Phase solubility studies

The phase solubility investigation was performed in order to find out the apparent stability constant (K) that represents the affinity of MA to the β-CD in water. For this, studies were carried out in triplicate following the Higuchi and Connors method^43^. Samples were prepared by mixing in sealed 50 ml Erlenmeyer flasks excess of MA (4.11 × 10^−3  ^g) with 30 ml of aqueous solutions containing successively increasing concentrations (0, 3, 6, 9, 12, 15, 18, and 21)×10^−3 ^M of β-CD. Then, the solutions were kept under agitation for 5 d at 25 °C. The solutions were then filtered through 0.45 μm filter pore size and the filtrate was assayed for drug concentration by ultra-violet spectroscopy (SHIMADZU UV-1800) at 282 nm where no absorbance due to the β-CD was observed.

### Determination of complex stoichiometry

The continuous variation method otherwise known as Job’s plot was used to ascertain the stoichiometry for MA:β-CD complexation^16^. The experiments were carried out in duplicate by mixing two equimolar solutions (10^−3 ^M) of β-CD (in water) and MA (in ethanol) in different molar ratio from 0 to 1 without variation of the final volume. After 7 d of stirring at 25 °C, solutions were analysed by ultra-violet spectroscopy at 282 nm. The absorbency changes (ΔAbs = Abs-Abs_0_) of MA in the presence (Abs) and absence (Abs_0_) of β-CD were plotted *versus R*, where *R*= [MA]/([MA] + [β-CD]). The Job’s plot showed a maximum at a specific molar ratio indicating the stoichiometry of the complexes.

### In silico molecular modelling studies

The molecular docking simulations were carried out in the Glide (grid-based ligand docking) application implemented in the Maestro 9.3 software (Schrodinger, LLC, New York, 2012)^44,^^45^. The crystal structure of β-CD was obtained from the Protein Data Bank PDB (ID: 1BFN). For adding the missing hydrogen, we used the Protein Preparation Wizard. Then, β-CD crystal structure was separated from α-amylase, followed by energy minimisation at RMSD convergence (0.30 Å with OPLS_2005 as a force field). In order to obtain possible MA conformers, the MA structure was designed using the Maestro structure builder and optimised with LigPrep tool. To get the appropriate ionisation state, LigPrep was run with the Epik option set version 3.7 (Schrödinger) to generate a possible state at pH 7.4. Finally, the geometrical optimisation was performed using the OPLS-2005 force field. The “Generate Grid” sub-application of the Glide tool allowed the generation of the grid by selecting the entire β-CD structure as the receiving site to locate the coordinates of the centre of the targeted receptor cavity. Then, the generated grid was configured as the MA docking receiver using the “extra-precision” (XP) flexible docking method, from the Glide tool. The binding affinity “ΔG” was calculated using the Prime MM-GBSA module version 4.5 (Schrödinger). The same work was done for the 2:1 MA:β-CD inclusion complex.

On the other hand, in order to study the MA:β-CD interaction in the 1:2 complex, the β-CD structure was extracted from the crystallographic parameters. This last was provided by the structural database system of the Cambridge Crystallographic Data Centre and optimised by minimising energy to get the most stable state. These results were obtained using the Materials Studio 6.0 software^44^. Then, the resulting structure was simulated in the Maestro 9.3 software^45^. We followed the same steps for the first two conjugates.

Once the docking search was completed, the conformations were applied with the best binding energy. The complex inclusion structures resulted from the docking calculations were computed with Materials Studio using the Dmol3 method with the database (B3LYP/6-31g (d, p))^46^^,^^47^. This last allowed the computation of the descriptor dielectric energy (solvation). We noted that this last provided more information on the solubility of inclusion complexes between MA with β-CD.

### Preparation of solid complexes

#### Solvent co-evaporation method

MA (0.723 g, 0.003c mol) and β-CD (1.700 g, 0.0015 mol) were dissolved in ethanol (100 ml), and distilled water (100 ml), respectively. Then, these solutions were mixed in a flask and stirred at 600 rpm for 2 h at 50 °C. The obtained clear solution was evaporated at 45 °C using a rotary evaporator (BÜCHI, rotavapor R-215) rolling at 100 rpm. The solid residue was further dried at 50 °C for 24 h and stored in bottles and kept in the refrigerator.

#### Kneading method

The calculated and exactly weighed amounts of β-CD (1.700 g) were wetted with a minimum water volume (1 ml) and mixed in a ceramic mortar to get a homogeneous paste. Then, MA (0.723 g) was progressively introduced; while KN, a small quantity of ammonium hydroxide (0.5 ml of 35% solution), was added to assist the dissolution of MA. The mixture was then blended for 1 h. During this process, a small quantity (1.5 ml) of water was added to the mixture in order to keep an appropriate consistency. The paste was dried in an oven at 50 °C for 24 h then grinded into a fine powder and stored in bottles and kept in the refrigerator.

#### Physical mixture

The PM was prepared by simple blending for 30 min in a ceramic mortar pulverised powders (0.723 g of MA and 1.700 g of β-CD). The resulting material was sieved and stored in bottles and kept in the refrigerator.

#### Percentage practical yield

The percentage practical yield helps in selecting the appropriate method of preparation and it gives efficiency of any method. So, these were determined to know about percent practical yield (PY) from the following equation^10^:

(1) $$\text{PY}\left(\%\right)=\frac{\text{Practical Mass}\left(\text{Inclusion complex}\right)}{\text{Theoretical Mass}\left(\text{Drug}+\text{Carrier}\right)}*100$$

#### Percentage drug extract

The percentage drug extract was determined by extraction of the MA from the complexes and its amount measured using a SHIMADZU-1800 UV–visible spectrophotometer. Therefore, a known amount of each complex (25 mg) was placed in a 25 ml volumetric flask, and ethanol was then added. The mixture was shaken for 5 h. Hence, the MA extractable amount was obtained through its UV photometric analysis (*λ*_max_ = 282 nm) using the standard curve of a bunch of known MA concentrations. The extractions were carried out in triplicate and the drug content in the complex was obtained using the following equation:

(2) $$\text{MA}\;\text{content}\;\text{in}\;\text{complex}\left(\%\right)=\left[\frac{\text{mass}\;\text{of}\;\text{MA}\;\text{extracted}}{\text{mass}\;\text{of}\;\text{complex}}\right]*100$$

### Characterisation of the ingredients and their complexes

#### Fourier transform infrared spectroscopy

The infra-red spectra of MA, β-CD, and their complexes were recorded with an IRAffinity-1S SHIMADZU spectrometer using the potassium bromide (KBr) disc technique (1% w/w of the samples in KBr). The scanning range was 4500–500 cm^−1^.

#### Differential scanning calorimetry

DSC analysis for MA, β-CD, and their inclusion complexes were carried out using a DSC SETARAM instrument. The samples (10–15 mg) were placed in sealed aluminium pans under nitrogen flow (20 ml/min) at a scanning rate of 10 °C/min, over the temperature range of 25–340 °C.

#### X-ray powder diffraction

X-ray powder diffractograms of individual components and those of complex systems were obtained on a PAN analytical kind X’ PERTPRO diffractometer. The radiation used was generated by a copper filter, wavelength 1.54 Å at 40 kV and 30 mA. Glass slide was covered with the sample to be analysed and scanned over a 2*θ* range from 10 to 40°, using a scan rate of 1°/min and a step scan of 0.02°.

#### Nuclear magnetic resonance (NMR)

The ^1^H and ^13 ^C NMR spectra were recorded at 400 MHz on a Brücker DRX 400 spectrometer in DMSO-*d*_6_. Chemical shifts are expressed in ppm (δ) downfield from internal tetramethylsilane (TMS). The NMR spectra were processed and analysed by MestReNova software 11.02.18153.

#### Scanning electron microscopy

The surface morphology of MA, β-CD, and binary complexes were captured by a scanning electron microscope (JSM 6360 A, JOEL) equipped with secondary electron detector. The samples were examined at an accelerating voltage of 10 kV.

### In vitro drug release test

The *in vitro* release test of pure MA and its complexes was performed using the United States Pharmacopoeia Paddle Method (Apparatus II) on Heidolph RZR 2041. Samples equivalent to 50 mg of MA were placed into a hard gelatine capsule, and then soaked into 900c ml of the simulated gastric medium (0.1c M HCl, pH 1.2) for 2 h. The dissolution media was maintained at 37 ± 0.5 °C and stirred at 100 rpm. At suitable time intervals, 3 ml of the dissolution medium was withdrawn, using a syringe, and filtered through 0.45 μm nylon disc filter. Then, an equivalent volume of fresh medium was added in order to maintain sinking conditions. The MA content was determined at 282c nm using an UV–VIS spectrophotometer. We noted that each dissolution test was carried out in duplicate. The kinetics of the MA released from inclusion complexes were determined by fitting the release profiles to the first order (3), Korsmeyer–Peppas (4) and Higuchi (5) theoretical models:

(3) $$F_{t}=1-\;\text{exp}\;\left(-K_{1}\cdot t\right)$$

where *F_t_* is the fraction of drug dissolved in time *t* and *K*_1_ is the first order release constant.

(4) $$\frac{M_{t}}{M_{i}}=K_{\text{KP}}t^{n}$$

where *M_t_*/*M_i_* is the fractional release of drug into the dissolution media, M_t_ is the release accumulation, and *M_i_* is the initial drug amount. *K*_KP_ is the Korsmeyer*–*Peppas constant, and *n* is the release exponent indicative of the drug release mechanism.

(5) $$F_{t}=K_{\text{H}}t^{0.5}+a$$

where *K*_H_ is the Higuchi release constant and a is a constant characterising the initial drug release.

Then, the selection of the best fit model is based on the regression coefficient value *R*^2^ which should be close to one^48^.

### In vitro anti-inflammatory activities

#### Protein denaturation method

The binary inclusion complexes were evaluated by using inhibition of bovine serum albumin (BSA) denaturation technique. This assay was done according to Mizushima and Kobayashi^49^ with minor modification. The MA drug and tested complexes (1 ml) containing 100 µg/mL of drug were mixed with 1 ml of 1% w/v BSA in phosphate buffer (pH 6.4) and incubated at 27 ± 1 °C for 15 min. The denaturation was induced by maintaining the reaction mixture at 75 ± 1 °C in a water bath for 10 min. Then, after cooling, the turbidity was measured at 660 nm (λ_max_ BSA). The percentage inhibition of denaturation can be calculated from reference where no drug was added. We noted that each experiment was done in triplicate and an average value was taken.

#### Membrane stabilisation method

The binary inclusion complexes were assayed by using human red blood cell (HRBC) membrane stabilisation method^50^. For this, fresh whole human blood (10 ml) was collected and transferred to heparinised centrifuge tubes. The tubes were centrifuged for 5 min at 3000 rpm, and washed three times with an equal volume of normal saline. The volume of the blood was measured and reconstituted as a 40% v/v suspension with isotonic buffer solution (10 mM sodium phosphate buffer pH 7.4). The activity of MA from all drug-carrier systems was analysed at the concentration of 100c µg/ml with the same dose of standard drug of pure MA.

## Results and discussion

### Phase solubility studies

**Figure 1.**
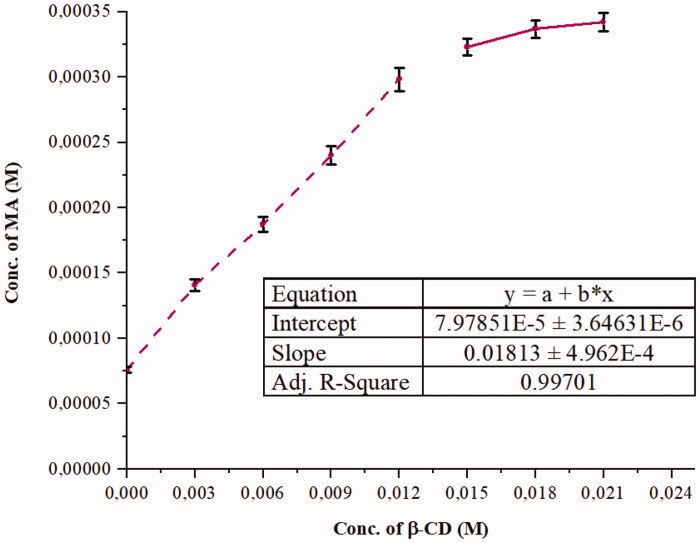
Phase solubility diagram of MA with β-CD in aqueous solution at 25 °C.

The phase solubility diagram of MA with various concentrations of β-CD in water is illustrated in Figure 1. β-CD formed A_N_-subtype complexes with MA. We note that the MA solubility increases with the β-CD concentration. However, a negative curvature occurs at higher CD concentrations (15–21 mM). This fact implies that β-CD is proportionally less effective at higher concentrations. This may occur as a result of the change of physical properties of the solution at higher β-CD concentration and the self-association of free β-CD molecules, thereby reducing the available concentration of free β-CD^15^. It should be noted that other researchers^40^^,^^42^ revealed A_L_ type phase solubility of MA:β-CD where the used β-CD concentration was lower than 1 0^−2 ^M.

Similar phase solubility profiles (curvature shapes) have been found by Pradines et al.^51^, for an antiparasitic drug complexed with a methyl-β-CD in water. Buchanan et al.^52^ also reported other similar profiles for some antifungal drugs and hydroxybutenyl-β-CD in some buffer. Equivalently, Rudrangi et al.^53^ presented similar profile for indomethacin and methyl-β-CD in phosphate buffer (pH 7.4) solution.

### Determination of complex stoichiometry

**Figure 2.**
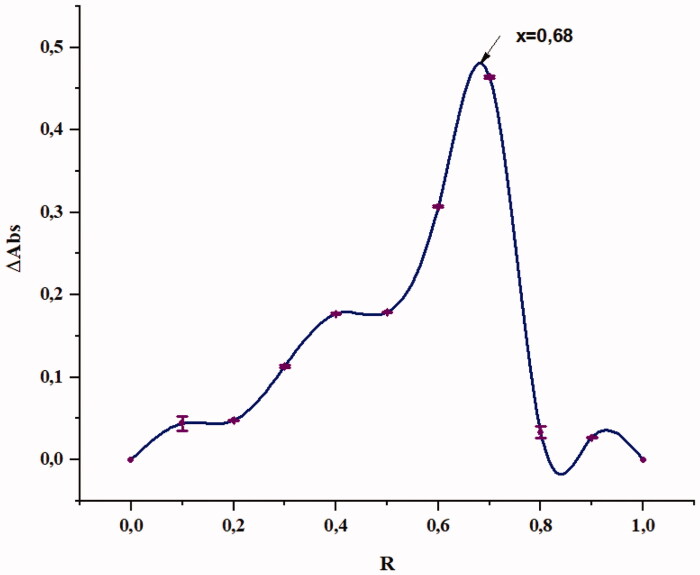
Continuous variation plot (Job’s plot) for the complexation of MA/β-CD (25 °C).

Job’s continuous variation technique is applied to determine the stoichiometry by utilising the absorption spectral data. As seen in Figure 2, the maximum ΔAbs variation was observed at mole fraction value of 0.67. This suggests a 2:1 stochiometric ratio of MA:β-CD.

### In silico molecular modelling studies

**Figure 3.**
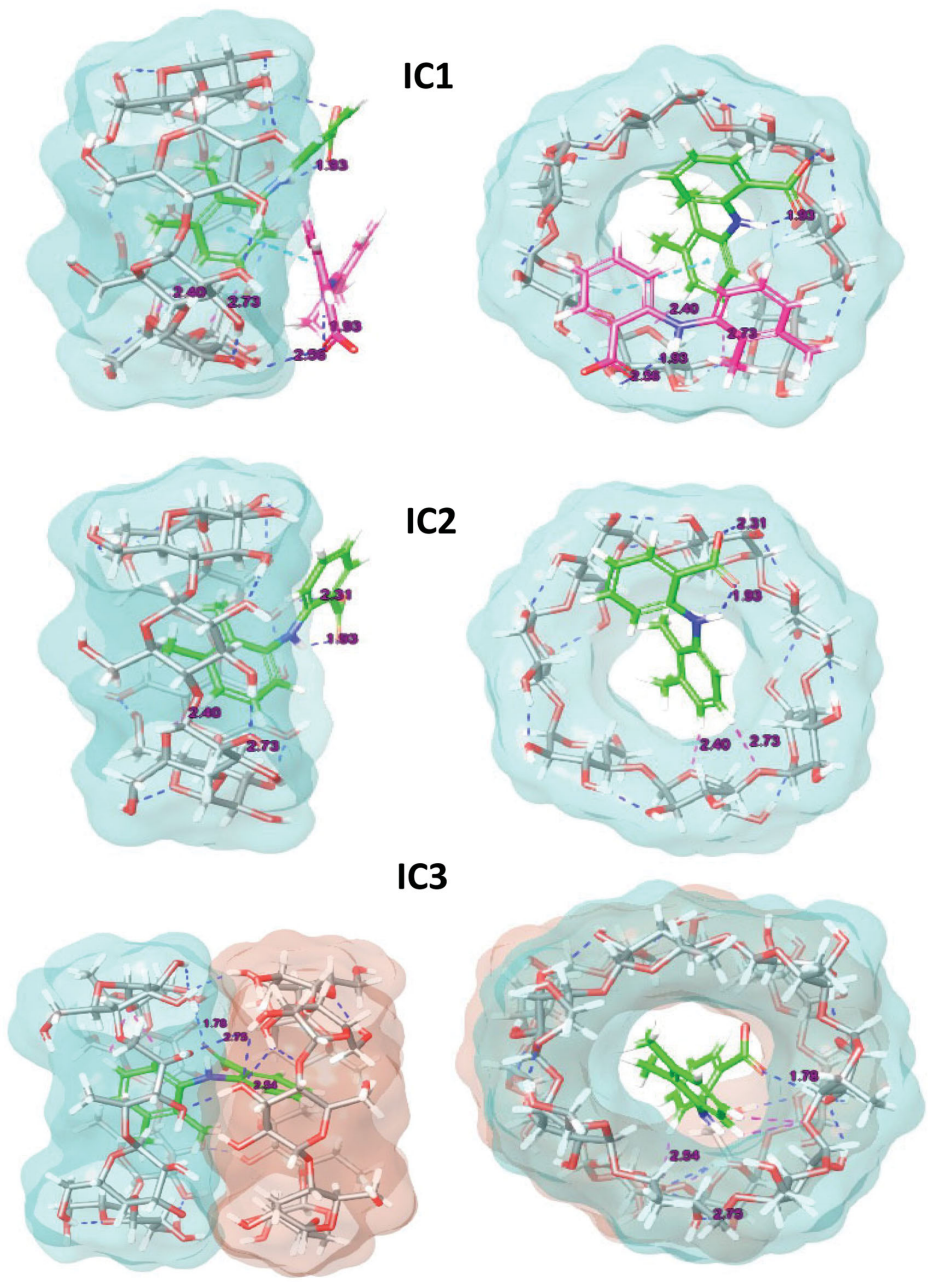
Docking binding poses of the inclusion complexes.

The molecular docking was used to explore the interaction of β-CD with MA in the inclusion complexes with different stoichiometry. The best pose of MA in inclusion complexes is illustrated in Figure 3.

The binding affinity expressed in the form of Glide docking score for the MA with β-CD is given by −7.890 kcal.mol^−1^ for IC1. Actually, this value is comparable to that of the IC3 (−7.375 kcal.mol^−1^), where the IC2 presents the lowest docking score. However, for complex stabilisation, contribution (from Van der Waals interaction) it takes higher value compared to lipophilic and hydrogen bonding interactions for all binary complexes (Table 1).

**Table 1.** Prime MM-GBSA calculations.

ΔG values in Kcal.mol^−1^						
Ratio (MA:β-CD)	Bind^a^	Docking score	Glide Lipo^b^	Glide vdw^c^	Glide Hbond^d^	Glide Emodel
1:1 (IC2)	−21.468	−4.575	−2.179	−21.123	−0.042	−26.612
2:1 (IC1)	−37.698	−7.890	−3.454	−37.387	−0.202	−46.262
1:2 (IC3)	−35.995	−7.375	−3.139	−28.990	−0.160	−50.976

**^a^**free energy of binding (Glide energy); **^b^**free energy of binding from lipophilic binding; **^c^**free energy of binding from van der Waals energy; **^d^**free energy of binding from hydrogen bonding.

**Figure 4.**
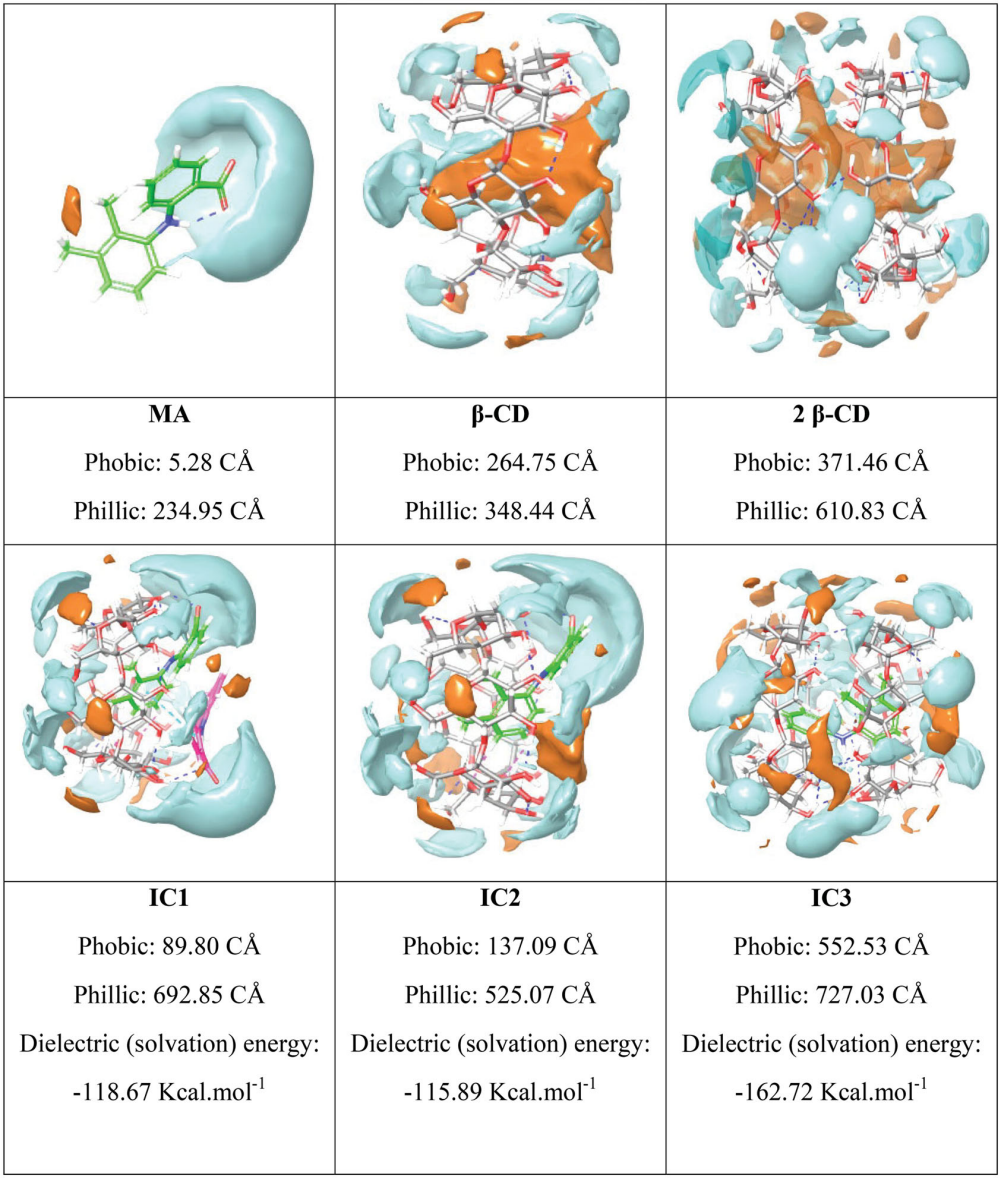
Hydrophobic (brown) and hydrophilic (blue) surface area of MA, β-CD and MA:β-CD binary inclusion complexes (CÅ, cubic Angströms).

On the other hand, Prime MM-GBSA module version 4.5 (Schrödinger) is used to obtain the binding affinity (ΔG) that represents the free energy change upon formation of the complex, in comparison to total individual energy based on change in the solvent-accessible surface area. It allows the stability determination of binary inclusion complexes^54^. In fact, the ΔG binding energy exhibits a similar behaviour as that of docking score calculations. The IC1 is the most stable complex (−37.698 kcal.mol^−1^) followed by IC3 (−35.995 kcal.mol^−1^), and IC2 (−21.468 kcal.mol^−1^) (Table 1). The introduction of a second MA molecule in the primary complex will enhance stability (Figure 3). This is a consequence of further hydrogen electrostatic interaction and improved filling of the β-CD cavity. Figure 4 provides more details about the hydrophobic and hydrophilic surface areas of MA, β-CD, and the binary complexes. From Figure 4, the hydrophilic area increases upon formation of IC1. In fact, higher polar surface area of the supramolecular inclusion complex improved the MA solubility.

### Preparation of solid complexes and loading

According to job’s plot results and the molecular docking observations (2:1) MA:β-CD complexes were prepared by PM, KN, and CE methods. In all inclusion methods, loss of complexes’ mass was observed. In CE method, large volumes of organic solvent and longer process cause more important loss of mass. So an experimental yield was calculated (Table 2) for CE. With an experimental loading value of 28.96%, the ratio of (2:1) MA:β-CD complex was also confirmed.

**Table 2.** Experimental yield and drug extraction from CE binary inclusion complex.

Complex	CE
Experimental yield (%)	90.1
Drug content (%)	28.96 ± 0.39
Experimental molar ratio MA:β-CD	1.9:1

### Characterisation of the ingredients and their complexes

#### Fourier-transform infrared spectroscopy

**Figure 5.**
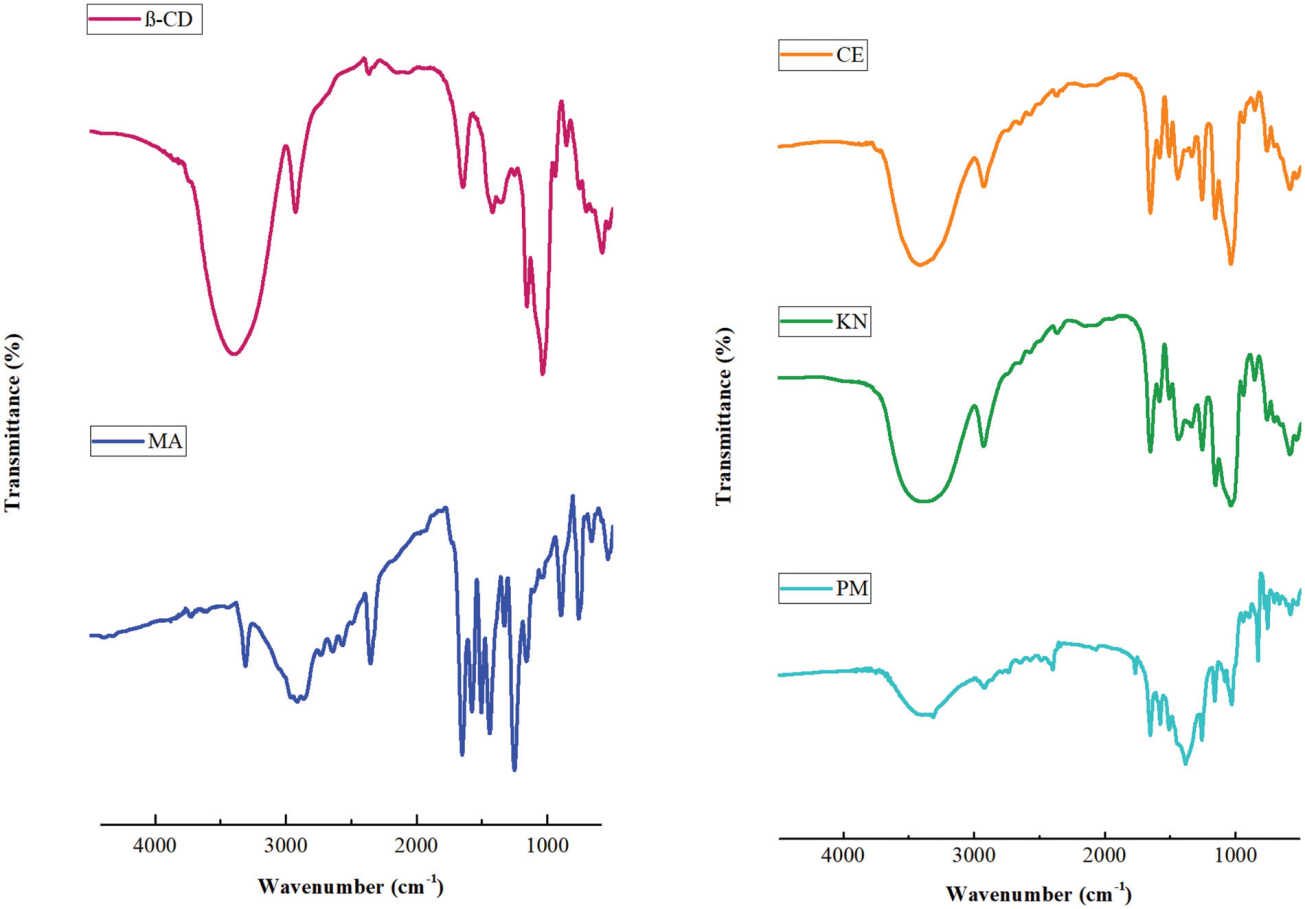
FT-IR spectra of MA, β-CD, and MA:β-CD binary inclusion complexes.

In order to examine the plausible interactions between MA and β-CD in the solid state, the IR spectra of binary complexes are compared to those of the PM and the pure drug (Figure 5). The spectrum of pure drug shows many intense and sharp absorption bands. Actually, this fact is due to the different functional groups existing in MA, for instance: aromatic ring, carboxylic group, mine group, and methyl group. In fact, the band recorded in the high wave numbers’ region can be used to distinguish the polymorph forms of MA. This very weak band at 3310 cm^−1^ is assigned to N − H stretching modes of the most stable polymorphism form I of MA^55^. The band at 2915 cm^−1^ is attributed to the ν(O − H) out-of-phase mode. Very intense and sharp band (recorded at 1649 cm^−1^) is due to stretching mode ν(C = O) of the carboxylic group. The bands due to the ν(C–C) stretching modes of the aromatic rings are recorded between 1500 and 1450 cm^−1^, where the band due to the deformation mode δ(N–H) of the amine group is recorded at 1574 cm^−1^. The bands for the methyl group δ(CH_3_) are recorded between 1470 and 1430 cm^−1^ and the band originated from the ν(C–N) stretching mode is recorded between 1160 and 1250 cm^−1^. Bands due to out-of-plane deformations, δ(N–H) and δ(C–H), are recorded below 1000 cm^−1^^55^.

FTIR spectrum of β-CD (Figure 5) shows that prominent absorption band at 3393 cm^−1^ is due to the O-H stretching vibration. A further peak appearing in the region of 2800–3000 cm^−1^ represents the stretching vibrations of CH and CH_2_ groups. The peaks appearing at 1022 and 1162 cm^−1^ can be assigned to the stretching vibrations of C-OH and C-O-C groups of β-CD. The peak at 750 cm^−1^ also represents the pyranose ring vibration^56^. Finally, the peak at 650 cm^−1^ is due to the presence of water in β-CD cavity.

The FTIR spectrum of the PM imitates both peaks of MA and β-CD, which could be considered as simple superimposition of MA and β-CD spectra. Therefore, the presence of chemical incompatibility among pure MA and β-CD is ruled out.

However, in the FTIR spectra of complexes prepared by CE and KN, MA bands are almost masked by the very intense and broad β-CD bands. The results indicate interactions of MA into the β-CD cavity. We notice that the CO band of MA at 1649 cm^−1^ is shifted to 1656 cm^−1^ in CE spectrum and to 1647 cm^−1^ in KN spectrum. Also, the N-H band at 1574 cm^−1^ is 1586 cm^−1^ in CE spectrum and 1577 cm^−1^ in KN spectrum. This is a usual phenomenon observed by researchers in synthesising the inclusion complexes between β-CD and a guest molecule^38^^,^^57^^,^^58^.

We can add that the inclusion of the MA molecule with its aromatic rings into the electron rich cavity of the β-CD may amplify the density of the electron cloud. This last can lead to an increase in energy and consequently higher IR frequency absorption. The hydrogen bonding contacts and Van der Waals forces in complexes could also alter the microenvironment and eventually could decline the frequency between the inclusion complex and its constituent molecules^57^.

The broad hydroxyl band of pure β-CD at 3393 cm^−1^ is shifted to higher frequency region in the FTIR spectra for the KN and CE complexes. This last can be considered as a good indication of the inclusion complex formation. Additionally, the aromatic C-H and N-H deformation bands for binary complexes strongly drifted towards lower wave number. Overall, the binary inclusion complexes did not display any new IR peaks signifying that no chemical bonds are formed with the obtained complexes.

#### Differential scanning calorimetric analysis

**Figure 6.**
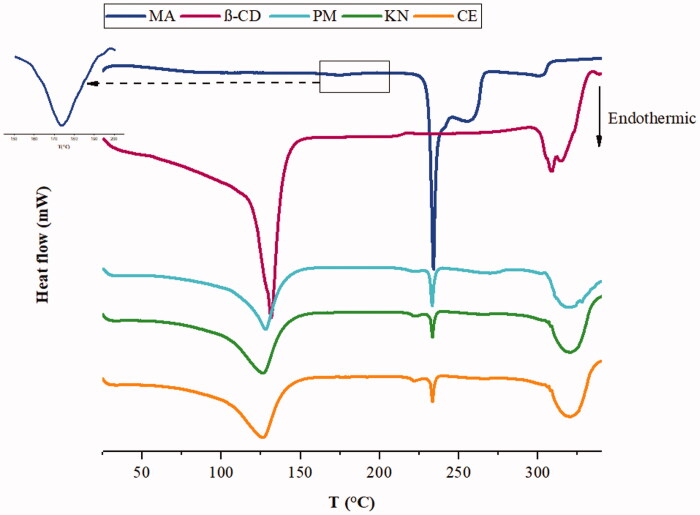
DSC thermograms of MA, β-CD, and MA:β-CD binary inclusion complexes.

The DSC technique is significantly important to understand the compatibility between the drug and CD in its complexes. When guest molecules are included in CD cavities, their melting, boiling, and sublimation points can shift to different temperatures or disappear^18^. The DSC thermograms for MA, β-CD, and binary systems are depicted in Figure 6.

The DSC thermogram of MA shows two endodermic peaks at 174 and 232 °C which correspond to the transition from form I–II and to the fusion of form II, respectively. MA decomposes after fusion and completely decarboxylates at 300 °C^59^. The thermal curve of β-CD shows a loss of physical water starting from 50 °C with a strong endotherm at 130 °C, which is caused by the liberation of crystal water molecules from the cavity. However, the peak corresponding to the decomposition process of β-CD is observed around 310 °C^60^. The thermal curves of the complexes show the endothermic peaks of the two individual components at their corresponding temperatures. This fact indicates the absence of chemical interaction between them. The thermogram of binary inclusion complexes illustrates the characteristic endothermic peak of the drug with reduced sharpness and intensity as compared to the pure drug, indicating an incomplete inclusion of the drug in the β-CD cavity. Hirlekar et al.^61^ described that similar phenomenon previously. Furthermore, in the DSC curves of the inclusion complexes, a small decrease of the endothermic peaks corresponding to β-CD dehydration indicates that the water molecules are present in a low quantity in the internal cavity of β-CD. This may be caused by the replacement of water molecules in the cavity by MA molecules occupying the same space. This in turn referred to the formation of inclusion complexes^18^.

#### X-ray powder diffraction

**Figure 7.**
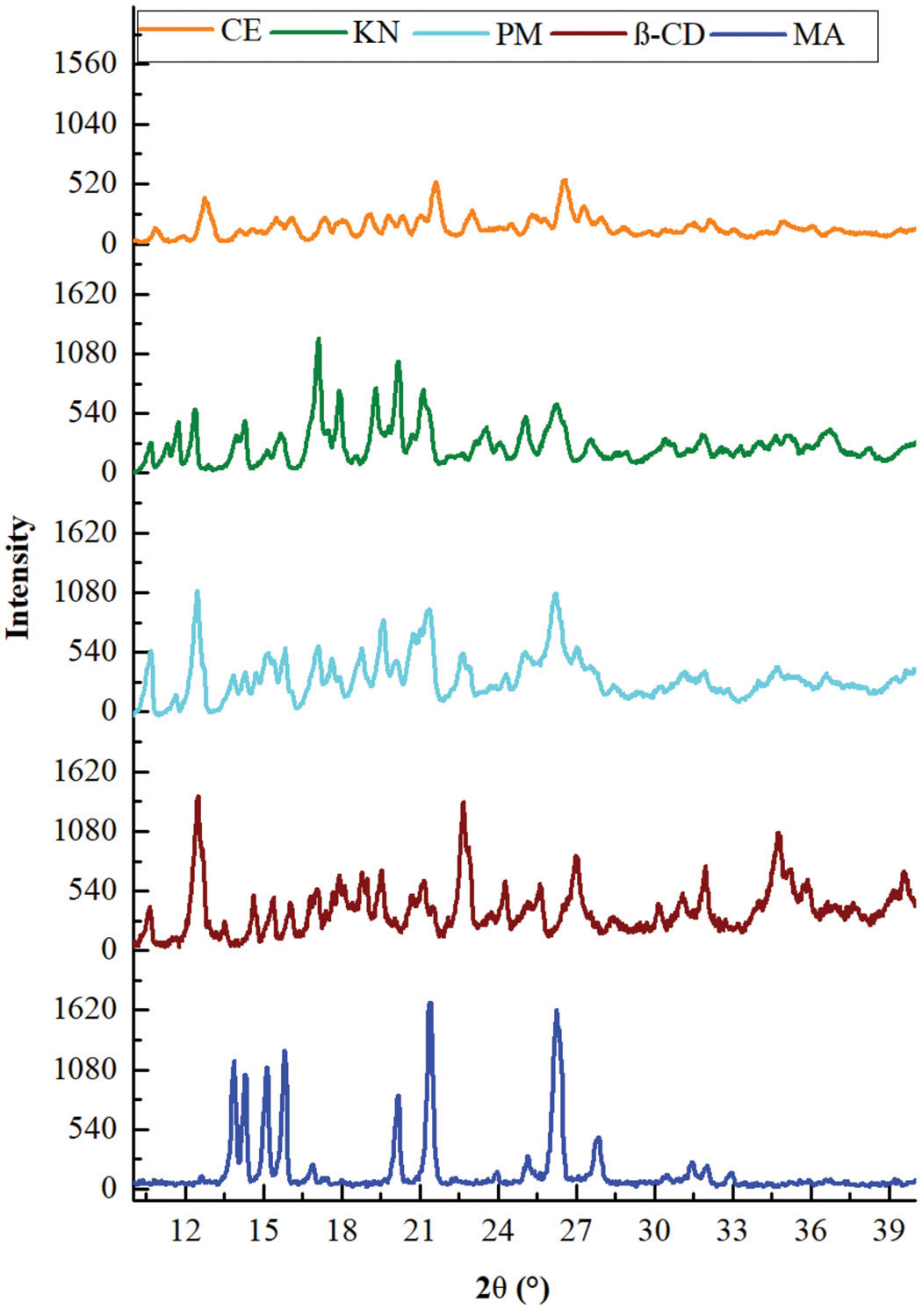
XRPD patterns of MA, β-CD, and MA:β-CD binary inclusion complexes.

XRPD is a useful tool for the detection of β-CD complexation in powder state. XRD studies could be employed to detect any change of crystallinity of a compound upon host–guest interactions.^29^ The X-ray diffraction patterns of MA, β-CD, and binary systems are presented in Figure 7.

MA showed characteristic peaks at 2*ϴ* equal to 13.8, 14.28, 15.8, 20.1, 21.4, 26.3, 27.7, and 32.8°. This corresponds to the I polymorph crystalline form of MA^55^. In addition, the diffractogram of β-CD displayed several sharp and intense diffraction peaks between 5° and 40° (2*ϴ*), which are indicative of its crystalline structure^18^, which corresponds to the well-known cage packing. The same diffraction peaks of MA and β-CD clearly appear in the PM, which indicates the absence of interaction between them. The binary PM shows also relatively less intense peaks of MA, but the crystallinity is evident. On the other hand, diffraction pattern of KN and CE complexes seems to be more diffused and the intensities of the characteristic peaks of MA are further reduced but still present. This suggests incomplete complexation between MA and β-CD leading to partial loss of the crystalline nature of MA. This observation is in accordance with the obtained results in the molecular modelling studies. Fernandes et al.^62^, while working with nicardipine-CD complexes, obtained similar results.

#### Nuclear magnetic resonance

The inclusion of a guest molecule in a host molecule is mainly based on intermolecular interactions (e.g. H bonds, van der Waals). NMR is a method of choice for studying such systems and the chemical shift analysis should indicate intermolecular interactions between MA and β-CD. ^1^H NMR spectrum of MA displays OH proton signal at 12.98 ppm (H-1) (Figure S1). The signal at 9.45 ppm is attributed to NH (H-2). The characteristic aromatic signatures are captured in the region 7.89–6.68 ppm (H-3-H-9). Sharp singlets corresponding to methyl protons (H-10 and H-11) are detected at 2.29 and 2.10 ppm, respectively. β-CD NMR spectrum shows signals at the δ value of 4.89 (H-1), 3.37 (H-2), 3.7 (H-3), 3.42 (H-4), 3.64 (H-5), and 3.76 ppm (H-6) (Figure S2). However, in order to confirm the inclusion of MA into β-CD, a comparison of the ^1^H NMR spectra of β-CD in the presence or absence of MA (Figures S3–S5) is necessary. Then chemical inclusion shifts (CIS) were calculated and listed in Table 3 (CIS with δ_guest_) and Table S1 (CIS with δ_host_). For complexes prepared by CE and KN methods, MA protons showed ^1^H-chemical shifts upon interaction with β-CD (Table 3). For example, all aromatic protons of MA (H-4, H-5, H-6 and H-7, H-8, H-9) are shifted, except for H-3. In addition, the NH function of MA also participates in the formation of the inclusion complex with a significant variation in CIS values (1.30 ppm for CE, 0.72 ppm for KN). Moreover, the chemical shift of ^1^H from COOH group in CE complex (CIS= −2.23 ppm) is the CIS value that has changed the most. All these variations clearly indicate that the guest–host interaction results in chemical shift changes. On the other hand, no shift was observed on all protons of MA when the PM method is used to get the corresponding inclusion complex. This last can be considered as a simple superimposition of MA and β-CD spectra. The overall analysis of CIS values for CH protons of β-CD also shows variations, both for the six protons of the three complexes (Table S1). By comparing the ^13^C NMR signals of the CE and KN complexes with MA alone (Figures S6–S10), large shifts of all carbon atoms of MA were observed (Table 4). As seen with ^1^H NMR spectra, the greatest changes are observed for the CE and KN complexes. The amplitude of the chemical shifts can reach values greater than 2, as for C-5 carbon (−2.76 ppm for CE, 2.22 ppm for KN).

**Table 3.** ^1^H NMR Chemical shifts (δ, ppm) for protons of MA alone (δ_guest_) and their complexation induced shifts (CIS = δ_complex_ – δ_guest_) in DMSO-*d*_6_ at 25 °C.

MA Protons	δ_guest_	CIS (CE)	CIS (KN)	CIS (PM)
H-1	12.98	−2.23	–	0
H-2	9.45	1.30	0.72	0
H-3	7.89	0	0	0
H-4	7.03	−0.21	0.05	0
H-5	7.12	−0.10	0.01	0
H-6	7.31	−0.18	−0.09	0
H-7	6.68	−0.08	−0.05	0
H-8	6.70	−0.09	−0.05	0
H-9	6.72	−0.09	−0.05	0
H-10	2.29	−0.03	−0.01	0
H-11	2.10	0.01	0.01	0

**Table 4.** ^13 ^C NMR Chemical shifts (δ, ppm) for carbons of MA alone (δ_guest_) and their complexation induced shifts (CIS = δ_complex_ – δ_guest_) in DMSO-*d*_6_ at 25 °C.

MA carbons	δ_guest_	CIS (CE)	CIS (KN)	CIS (PM)
C-1	170.66	–	0.34	0
C-2	149.22	−2.2	−1.43	−0.02
C-3	138.81	1.4	0.57	0
C-4	138.35	−0.93	−0.72	0
C-5	134.66	−2.76	−2.22	0
C-6	132.17	−1.16	−0.35	0
C-7	131.71	−2.64	−1.68	−0.02
C-8	129.89	−1.26	−1.08	−0.01
C-9	126.49	−2.22	−1.28	0.01
C-10	122.66	–	−2.31	−0.02
C-11	116.71	2.16	−0.60	0.01
C-12	113.55	2.43	−0.62	−0.01
C-13	111.69	1.11	–	0.03
C-14	20.69	−0.31	−0.38	0.01
C-15	14.14	−0.45	−0.45	0

In conclusion, the presence of H-bond interactions should lead to more stable complexes specially here for CE and KN complexes and then should increase the solubility of MA. Among the three methods investigated, the CE complex is the most stable complex prepared, with highest CIS values.

#### Scanning electron microscopy

**Figure 8.**
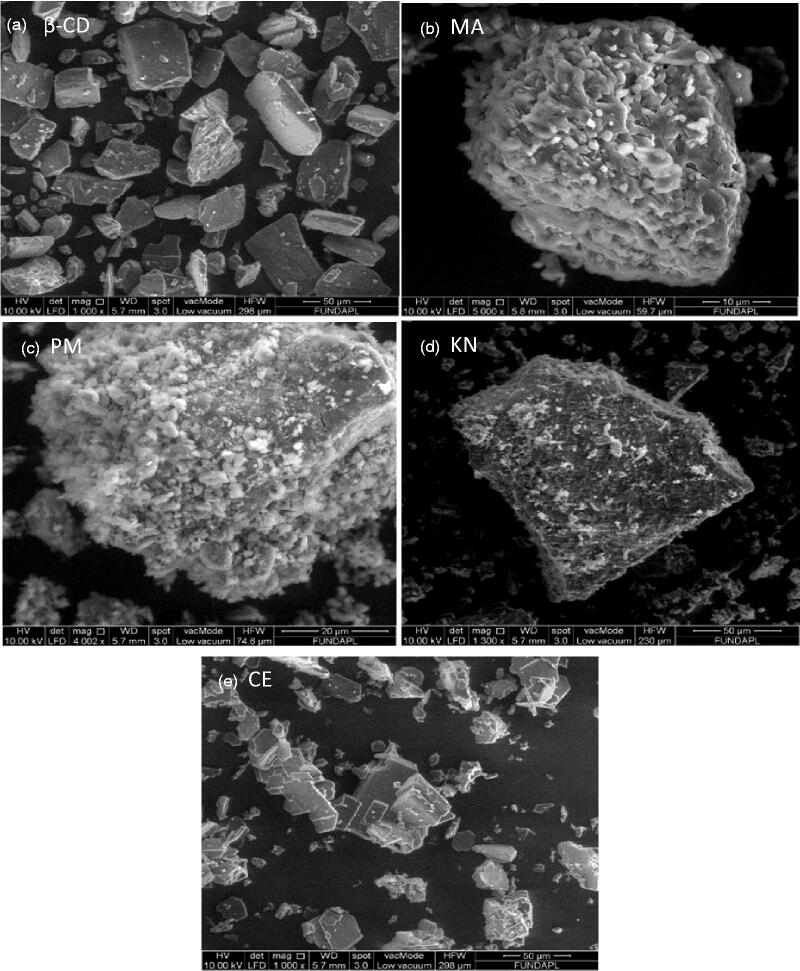
SEM micrographs of β-CD (a), MA (b), and MA:β-CD binary inclusion complexes (c-e).

Figure 8 presents the micrographs of MA, β-CD, and MA:β-CD binary systems prepared by various processing methods. The morphological changes may be used as an evidence for the interactions between molecules. β-CD (Figure 8(a)) exists as broken bricks, distributed in parallelogram, which are well separated from each other^63^. MA takes the form of flake crystalline particles (Figure 8(b)), irregularly sized with a tendency to self-agglomerate^64^. The PM method respects the original morphology of each component; the MA crystals are adhered to smooth surface of β-CD (Figure 8(c)). It is possible to distinguish a reduction in the agglomerated drug on the surface of β-CD in the case of KN product (Figure 8(d)) when compared with the PM complex. A significant change in the morphology of the inclusion complex was observed using CE method (Figure 8(e)). In fact, it is found that the crystal nature of MA disappeared. Micrographs show small, more agglomerated, and amorphous smooth structures, which suggest that MA molecules are well dispersed in the β-CD cavities. These results are consistent with the data obtained with the DSC and XRPD studies.

### In vitro drug release test

**Figure 9.**
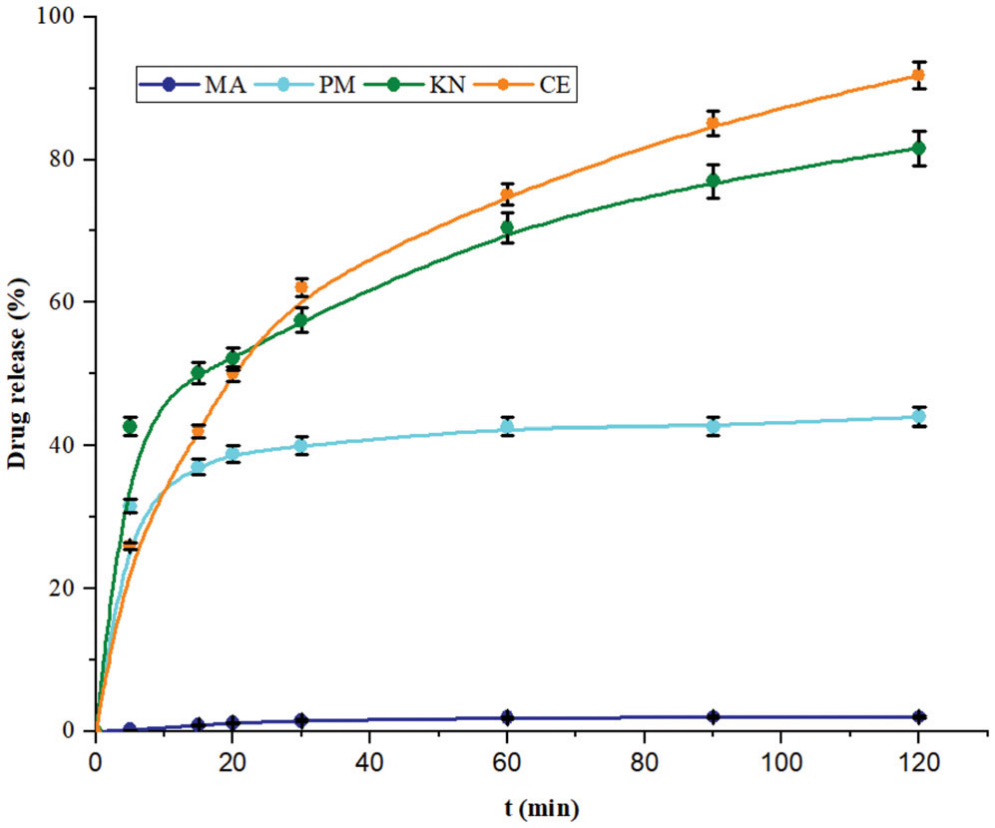
Dissolution profiles of MA and MA:β-CD binary inclusion complexes.

Figure 9 illustrates the dissolution profiles of MA from various (2:1) binary systems in 0.1 M HCl (pH 1.2). In fact, all the binary systems exhibit a more rapid release and a greater extent of dissolution compared to the drug alone. The most important enhancement of the drug dissolution properties is observed with the binary mixture prepared by CE, followed by KN and PM.

The improved MA dissolution characteristics of the PM binary system may be explained by the drug wettability enhancement at the early stages of dissolution process, due to the coexistence of drug and β-CD in the dissolution medium and/or the existence of interaction(s) between external β-CD cavity and MA. Indeed, because of the hydrophilicity of its outer surface, β-CD acts as a surfactant. Thus, it reduces the interfacial tension between the poorly soluble drug and the dissolution medium, resulting in a higher dissolution rate of the drug as proposed by Bera et al.^18^. Moreover, KN and CE binary systems showed a greater extent of dissolution than those of the pure drug and the PM. This enhancement may be due to partial trapping of the drug in β-CD verified by molecular modelling, FTIR, DSC, XRPD, and SEM experiments. It may confer a certain hydrophilicity and then may increase the solubility and wettability of MA. Furthermore, the reduced crystallinity of MA in the KN and CE binary mixtures is considered as an important point in the improvement of its dissolution. This could be related to the higher Gibbs free energy^65^ of the amorphous form.

In order to study the mechanism of release of MA from the different complexes, three kinetic models were used. The fitting results are summarised in Table 5. On the basis of the *R*^2^ values, the Higuchi model is the most appropriate model for the KN and signified that the mechanism of MA release from β-CD is governed by diffusion. The K_H_ release constant of the PM is the lowest (0.012 min^−1/2^), that confirmed no appreciable interaction between MA and β-CD. However, CE formulation presented a higher value of K_H_ release constant (0.074 min^−1/2^) which indicated the complex formation and the enhancement of drug dissolution compared to all formulations. In addition, the exponent (*n*) of the Korsmeyer–Peppas model indicated that the drug release is related to a quasi-Fickian diffusion since the values of *n* are lower than 0.5.

**Table 5.** Mathematical models of drug release kinetics: application to MA:β-CD binary inclusion complexes.

MA:β-CD	First order		Higuchi			Kosmeyer–Peppas			% Drug release after 120 min
	*R* ^2^	*K*_1_ (min^−1^)	*R* ^2^	*K*_H_×100 (min^−1/2^)	a	*R* ^2^	*K*_KP_	*n*	
PM	0.6553	0.0022	0.8363	1.2367	31.7150	0.9424	3.5851	0.0997	44.00
KN	0.9113	0.0054	0.9934	4.6504	32.2330	0.9784	3.4922	0.2154	81.60
CE	0.7551	0.0090	0.9688	7.4297	14.7730	0.9832	6.8865	0.3980	91.80

### In vitro anti-inflammatory activities

The last stage of our study was to evaluate the real potency of (2:1) MA:β-CD binary inclusion complexes *in vitro*. Since MA has marked anti-inflammatory activity, two assays were used, namely BSA denaturation and HRBC membrane stabilisation, to investigate the anti-inflammatory activity of studied complexes^48^^,^^49^. In certain inflammatory and arthritic diseases, protein denaturation leads to the production of autoantigen. In addition, neutrophils appear to be activated inappropriately and then release lysosomal enzymes that further promote inflammation like chemo-attractants (eicosanoids and chemokines) or cytokines. Additionally, due to the close similarity of the erythrocyte and lysosome membranes, stabilisation of erythrocyte membrane is considered as a preventive measure for the treatment of inflammation disorders. Then the prevention of hypotonicity-induced HRBC membrane lysis is a good marker for estimating the anti-inflammatory property of products^66^.

**Figure 10.**
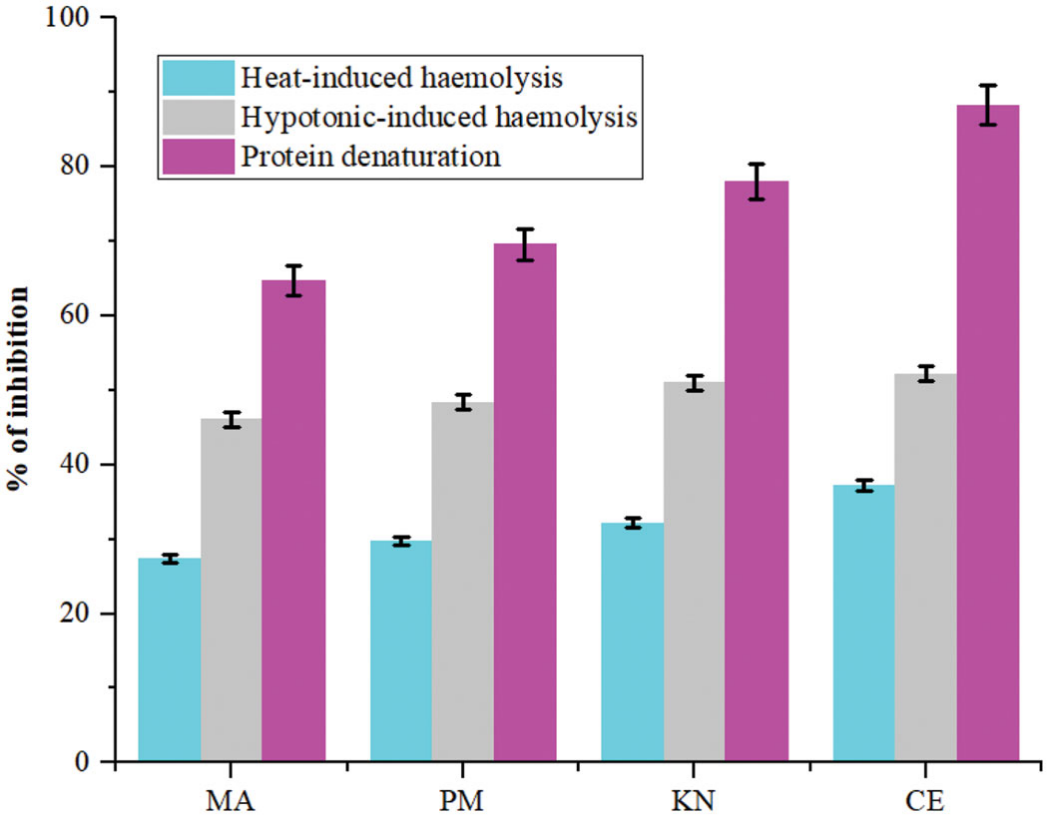
Comparison of the activity of MA and MA:β-CD binary inclusion complexes on BSA denaturation and HRBC membrane stabilisation.

The obtained results are shown in Figure 10. Inclusion complexes have considerably protected the BSA from denaturation (inhibition >69%). With the CE complex, the percentage rises up to 88%. This observation is well correlated to the rate of MA release (CE > KN > PM, see Figure 9). Additionally, the inclusion complexes are able to protect the membrane of erythrocyte from lysis induced by heat and hypotonicity. We also observed the best result with CE complex, the inhibition of heat-induced and hypotonic-induced haemolysis is equal to 37% and 52%, respectively.

## Conclusions

In this work, the phase solubility diagram and job’s plot experiment were used to determine the stoichiometry of the MA:β-CD complex. Then molecular modelling approach helped (i) to select the most stable inclusion complex (2:1), (ii) to determine intermolecular energy contributions, and (iii) to predict hydrophilic surfaces and drug solubility (e.g. solvation energy). Inclusion complexes of MA:β-CD in the 2:1 molar ratio were prepared using three methods, namely PM, KN, and CE. FTIR and NMR studies showed no evidence of chemical reactions between the drug and β-CD. DSC, XRPD, and SEM experiments confirmed partial amorphism of the MA after inclusion complexation indicating that MA was well dispersed in the β-CD cavities. These results suggest an enhanced dissolution profile compared to the crystalline form. All three formulations showed a significant improvement of the MA dissolution; however, the CE complex exhibited the highest *K*_H_ value. The CE method is thus the most appropriate method to get improved MA dissolution properties. Actually, the (2:1) MA:β-CD binary complex obtained by CE method constitutes an interesting alternative to formulate MA. Protein denaturation and membrane stabilisation assays also confirmed the therapeutic benefits of MA when used as (2:1) MA:β-CD complex. Finally, this approach of preparing inclusion complexes in an optimised ratio could allow other poor-water soluble NSAIDs to be studied again.

## Supplementary Material

Supplemental Material
