# Supplementary material for: Solubility enhancement of mefenamic acid by inclusion complex with β-cyclodextrin: in silico modelling, formulation, characterisation, and in vitro studies
Source: J Enzyme Inhib Med Chem. 2021 Feb 8;36(1):605–17. doi: 10.1080/14756366.2020.1869225 (PMC8759728; doi:10.1080/14756366.2020.1869225)
Supplement: Supplemental Material [file IENZ_A_1869225_SM2781.pdf]

SUPORTING INFORMATION

Solubility Enhancement of Mefenamic Acid by Inclusion Complex with β-Cyclodextrin: In Silico Modelling, Formulation, Characterization and In Vitro Studies

Dounia Sid[a], Milad Baitiche[a,*], Zineb Elbahri[b], Ferhat Djerboua[a], Mokhtar Boutahala[c], Zouhair Bouaziz[d] and Marc Le Borgne[d,e*]

[a]Laboratoire de Préparation, Modification et Applications des Matériaux Polymériques Multiphasiques, Département de Génie des Procédés, Faculté de Technologie, Université Ferhat Abbas Sétif-1, Boulevard du Sipion, Sétif 19000, Algérie; [b]Laboratory of Materials and Catalysis, Faculty of Exact Sciences, Djillali Liabès University of Sidi Bel Abbès, 22000 Algeria; [c]Laboratoire de Génie des Procédés Chimiques, Département de Génie des Procédés, Faculté de Technologie, Université Ferhat Abbas Sétif-1, Boulevard du Sipion, Sétif 19000, Algérie; [d]EA 4446 Bioactive Molecules and Medicinal Chemistry, SFR Santé Lyon-Est CNRS UMS3453 - INSERM US7, Université Claude Bernard Lyon 1, Univ Lyon, Lyon, 69373, France; [e]Small Molecules for Biological Targets Team, Centre de recherche en cancérologie de Lyon, Centre Léon Bérard, CNRS 5286, INSERM 1052, Université Claude Bernard Lyon 1, Univ Lyon, Lyon, 69373, France

* Addresses for correspondence: Milad Baitiche, baitiche_milad@yahoo.fr, Laboratoire de Préparation, Modification et Applications des Matériaux Polymériques Multiphasiques, Département de Génie des Procédés, Faculté de Technologie, Université Ferhat Abbas Sétif-1, Boulevard du Sipion, Sétif 19000, Algérie; Marc Le Borgne, marc.le-borgne@univ-lyon1.fr, Faculté de Pharmacie - ISPB, EA 4446 Bioactive Molecules and Medicinal Chemistry, Université Claude Bernard Lyon 1, 8 avenue Rockefeller, Lyon cedex 8, 69373, France

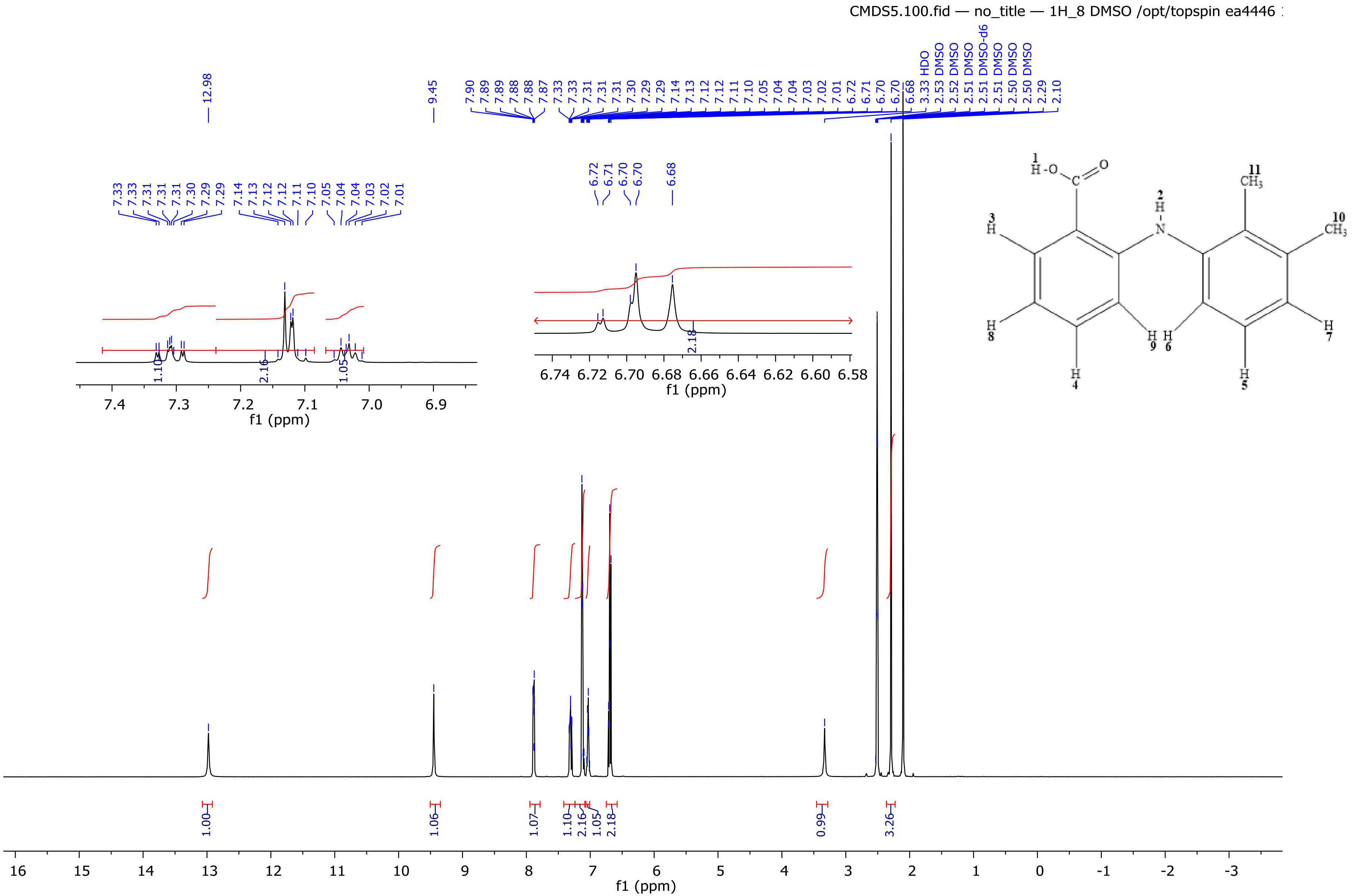

Figure S1. ^{1}H NMR Spectrum of MA.

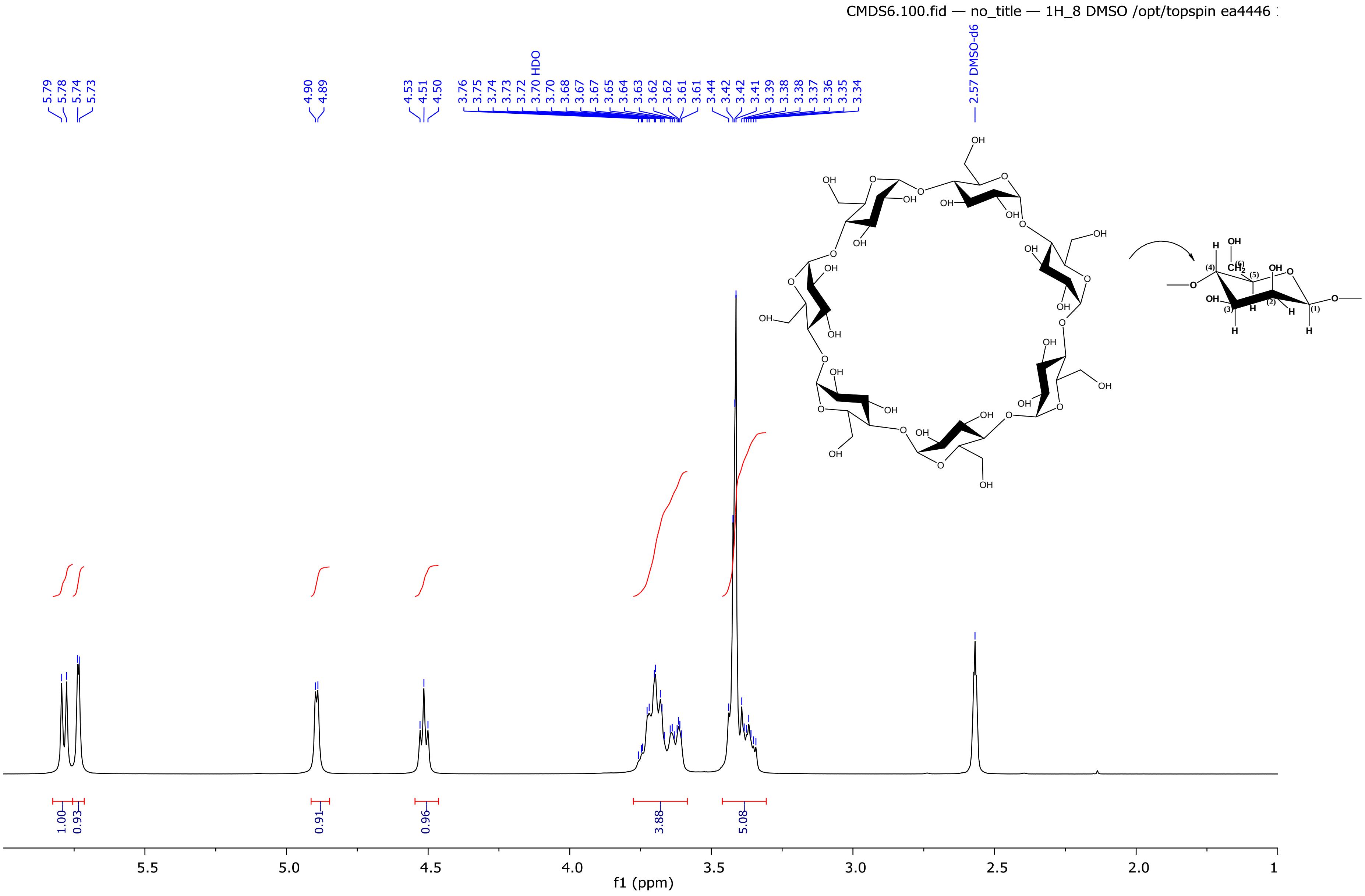

Figure S2. ^{1}H NMR Spectrum of β-CD.

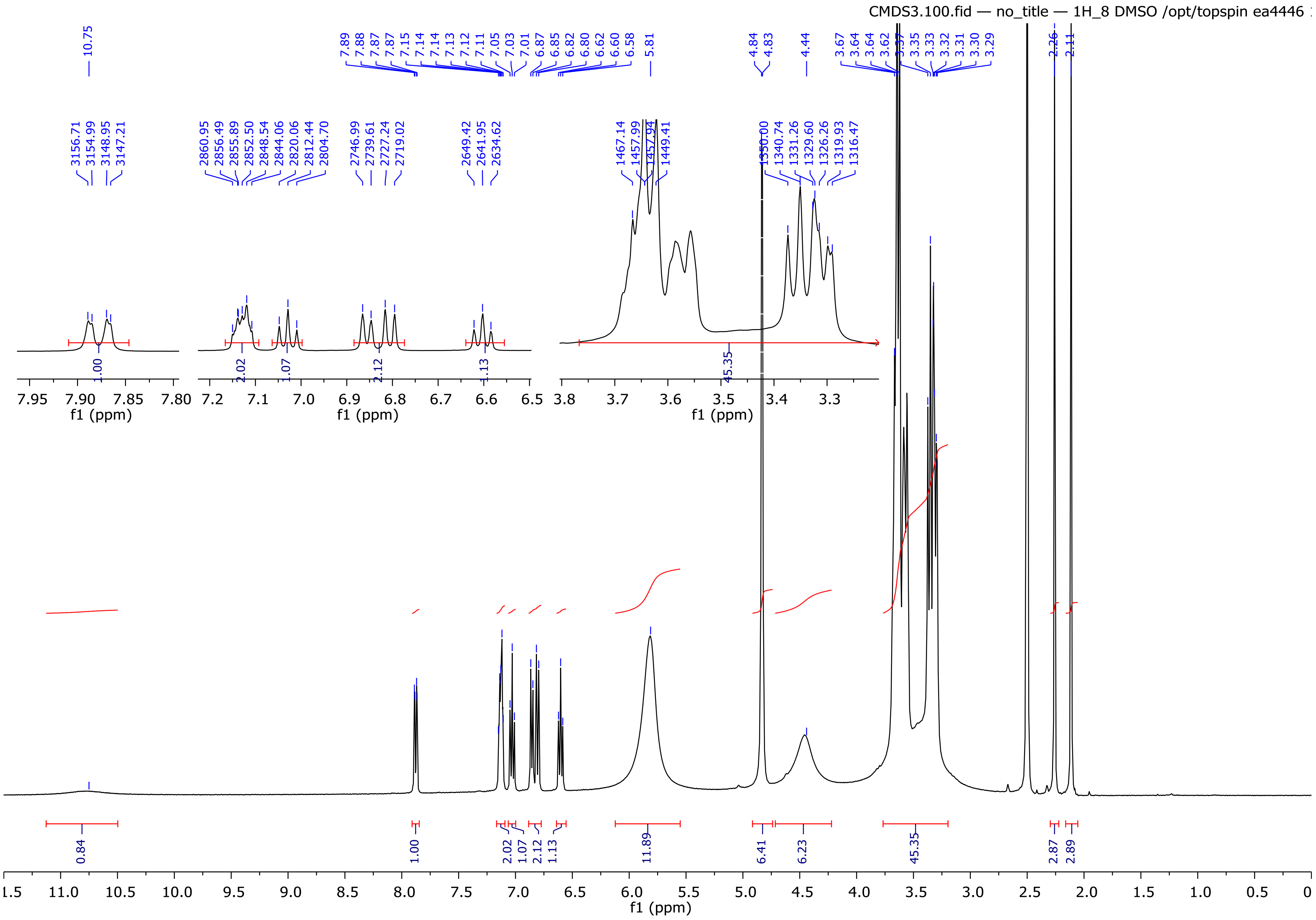

Figure S3. ^{1}H NMR Spectrum of CE inclusion complex.

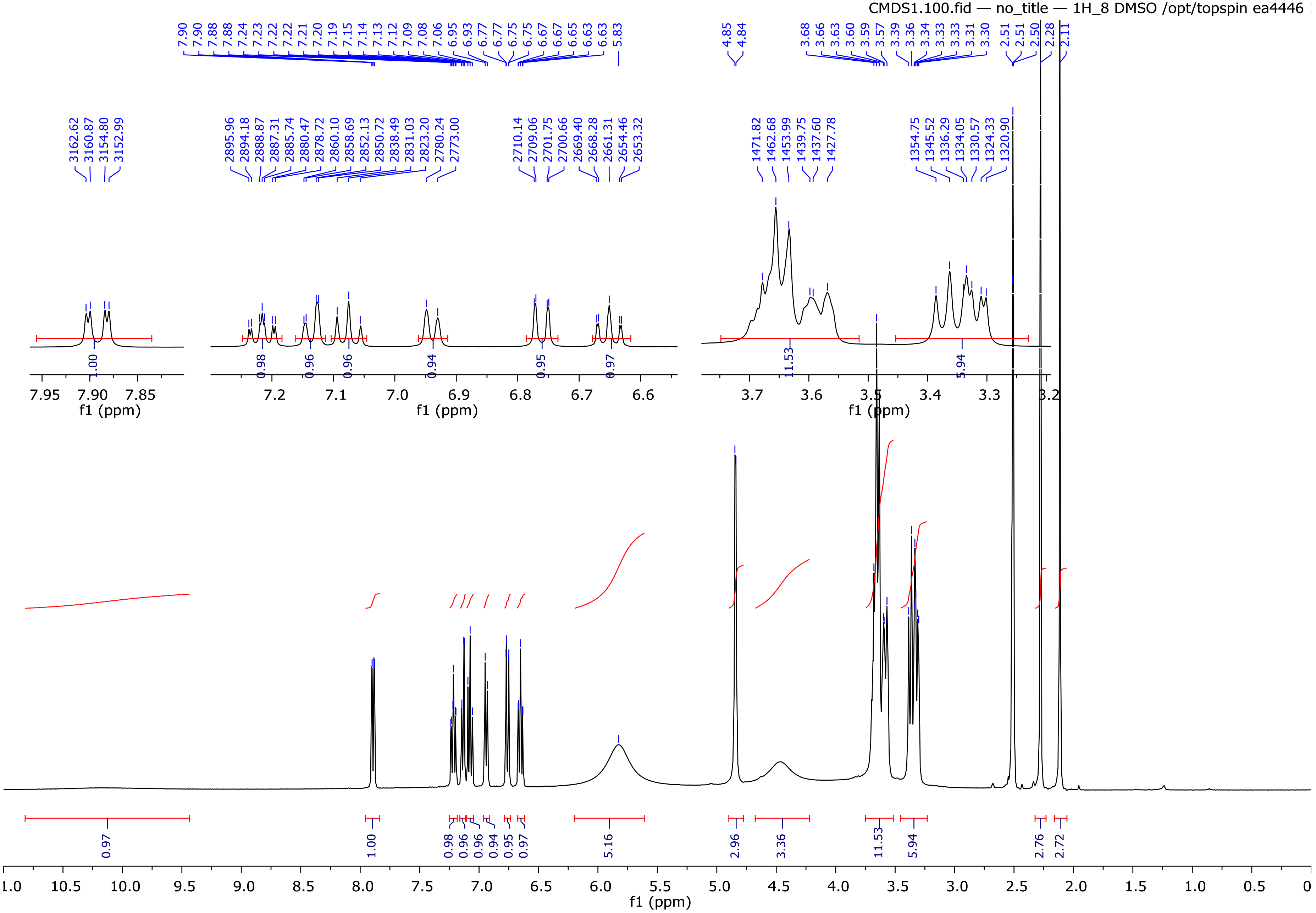

Figure S4. ^{1}H NMR Spectrum of KN inclusion complex.

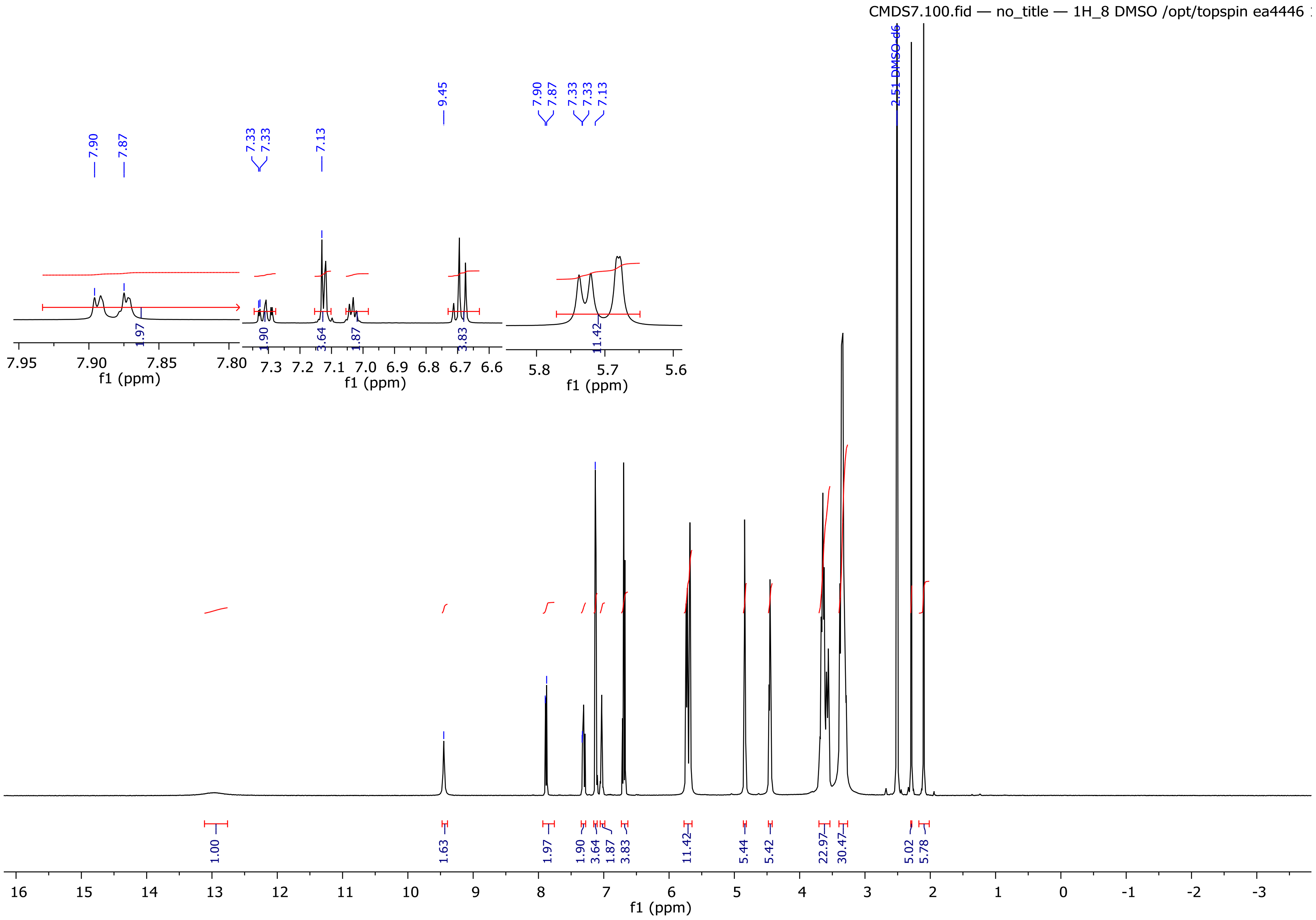

Figure S5. ^{1}H NMR Spectrum of PM inclusion complex.

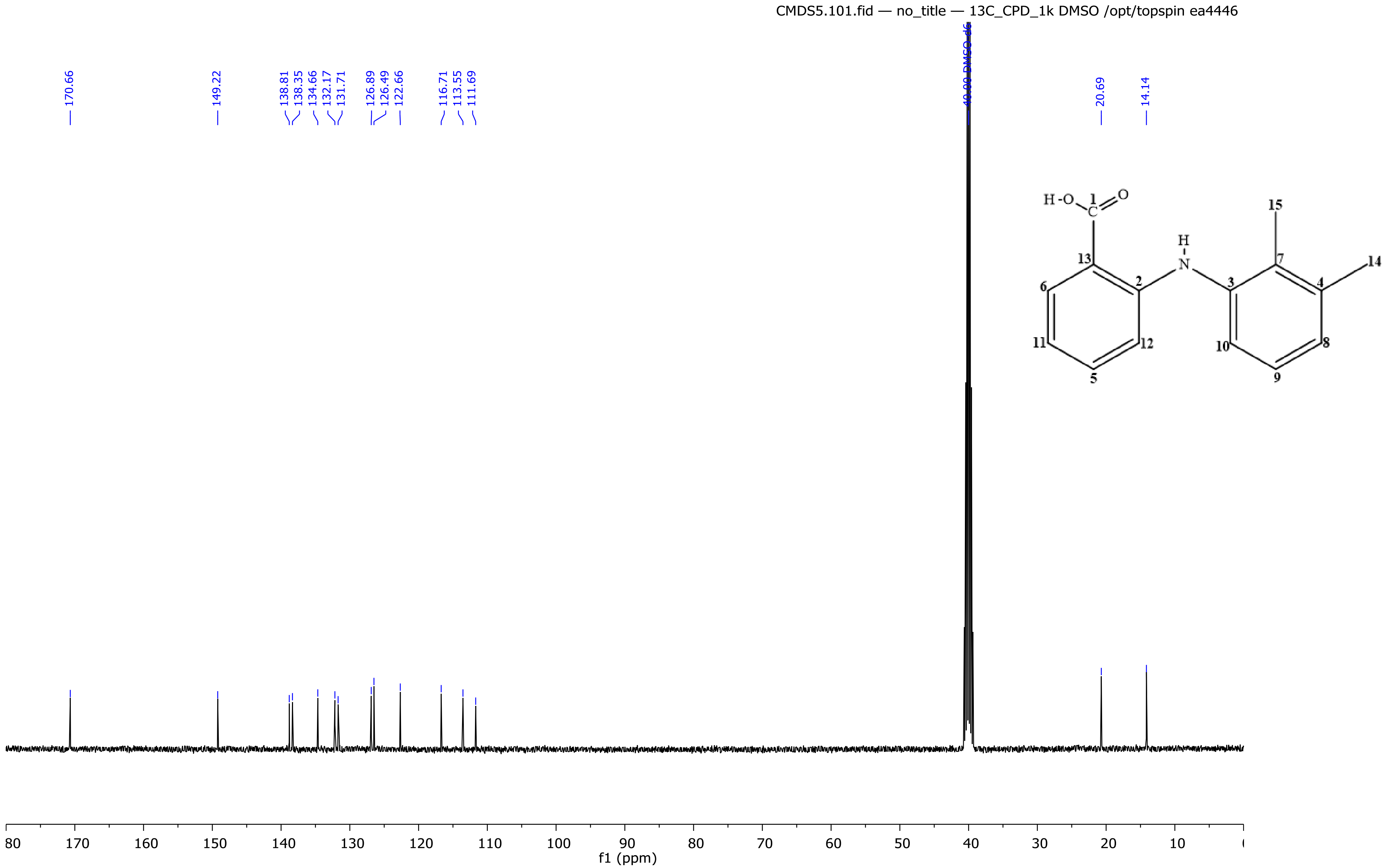

Figure S6. ^{13}C NMR Spectrum of MA.

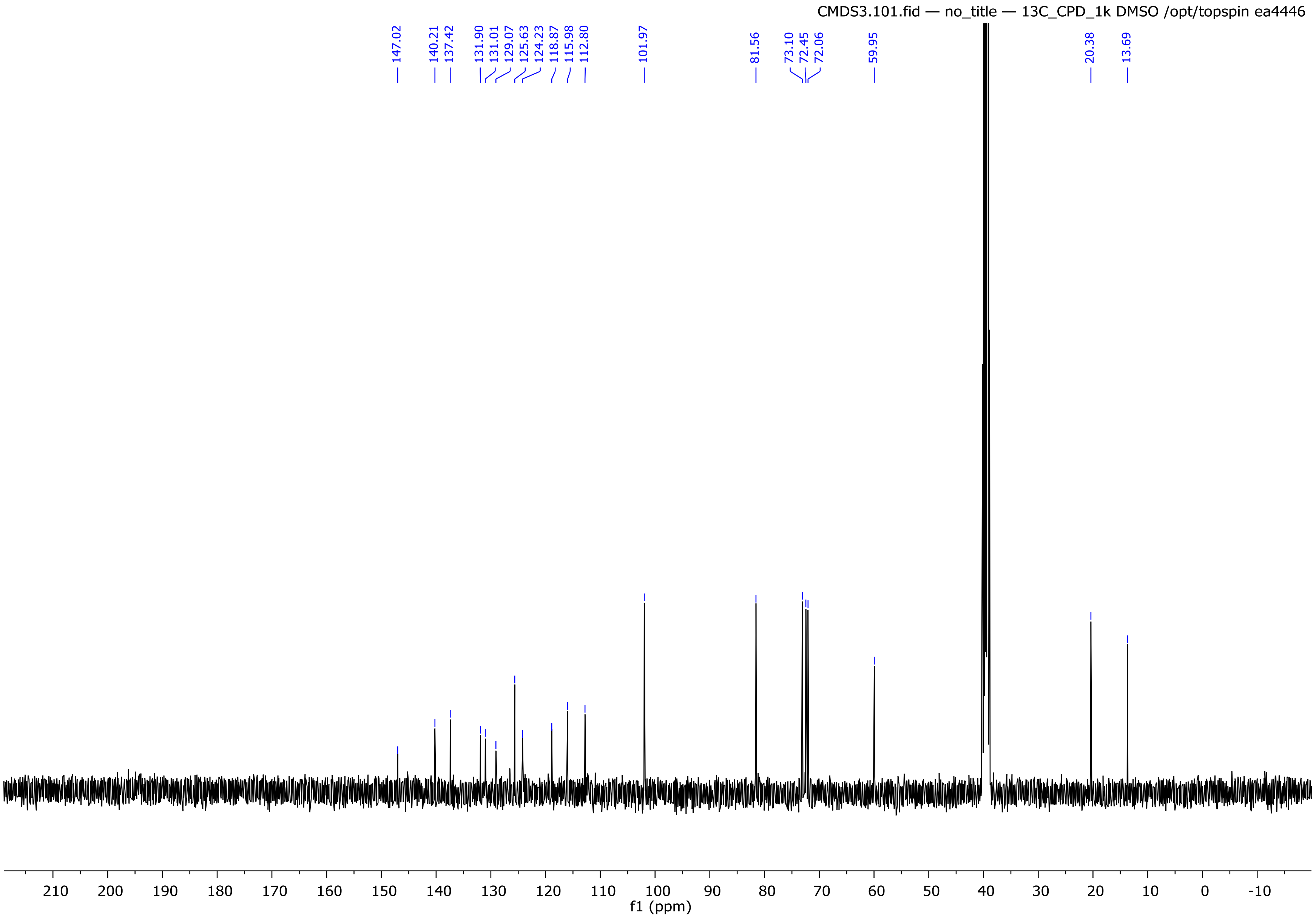

Figure S7. ^{13}C NMR Spectrum of CE inclusion complex.

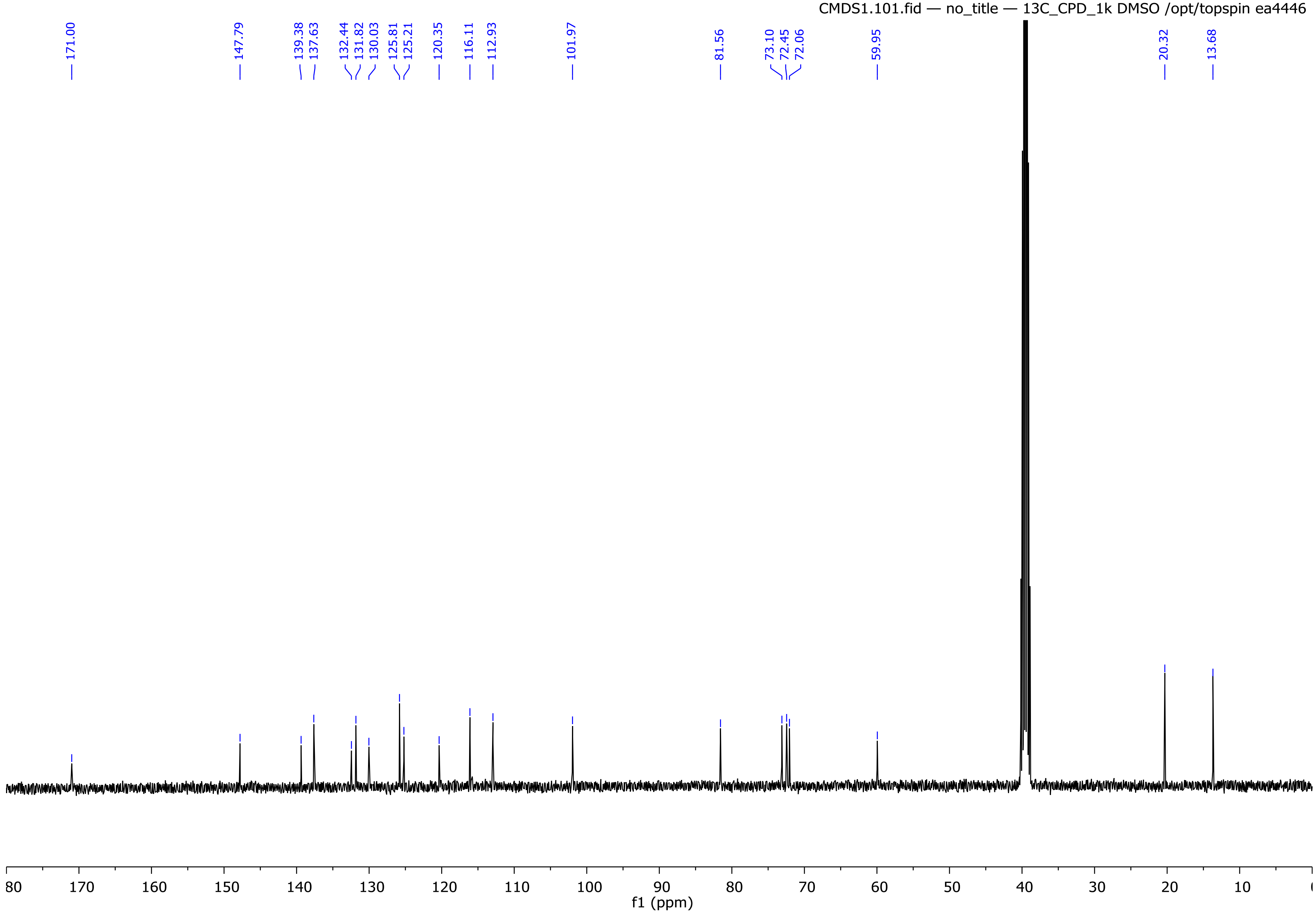

Figure S8. ^{13}C NMR Spectrum of KN inclusion complex.

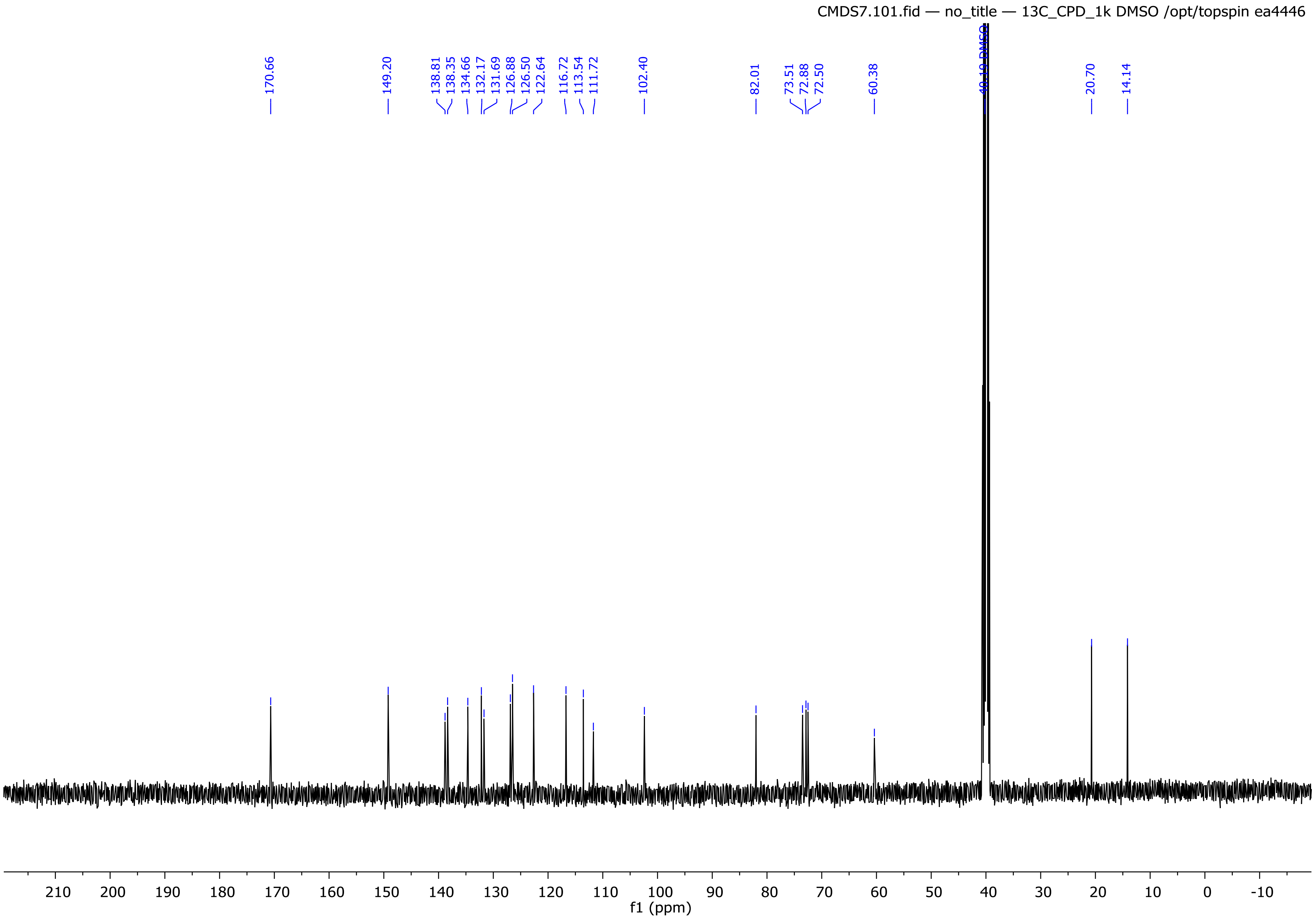

Figure S9. ^{13}C NMR Spectrum of PM inclusion complex.

Table S1. ^{1}H NMR Chemical shifts (δ, ppm) for CH protons of β-CD alone (δ_{host}) and their complexation induced shifts (CIS = $\delta_{complex} - \delta_{host}$) in DMSO-$d_6$ at 25 °C.

CH Protons of β-CD	δ_{host}	CIS (CE)	CIS (KN)	CIS (PM)
**H-1**	4.89	-0.05	-0.05	-0.06
**H-6**	3.76	-0.05	-0.06	-0.06
**H-3**	3.70	-0.05	-0.04	-0.05
**H-5**	3.64	-0.05	-0.04	-0.05
**H-4**	3.42	-0.06	-0.06	-0.06
**H-2**	3.37	-0.04	-0.04	-0.04
